# Measuring Asian hate: Discordant reporting of race-based hate incidents and unfair treatment and association with measures of wellbeing

**DOI:** 10.3389/fpubh.2022.958857

**Published:** 2022-10-10

**Authors:** Ninez A. Ponce, Alexander C. Adia, Rachel A. Banawa, Sean Tan, Melanie D. Sabado-Liwag

**Affiliations:** ^1^UCLA Center for Health Policy Research, Los Angeles, CA, United States; ^2^Department of Health Policy and Management, Fielding School of Public Health, University of California, Los Angeles, Los Angeles, CA, United States; ^3^Filipinx/a/o Community Health Association, Los Angeles, CA, United States; ^4^Department of Public Health, California State University, Los Angeles, CA, United States

**Keywords:** population surveillance/methods, mental health, survey measurement, interpersonal conflict at home, discrimination, anti-Asian racism, health access, public safety

## Abstract

**Background:**

During COVID-19, anti-Asian discrimination increased in attention. Hate and unfair treatment are related but do not completely overlap. We expect those who report a hate incident would also report race-based unfair treatment, yet feelings of social desirability or self-blame may lead to under-reporting of unfair treatment.

**Objectives:**

To describe reporting of an experience of race-based hate but not an experience of race-based unfair treatment among Asians in California and explore the association between this reporting discordance with (1) serious psychological distress, (2) forgoing needed medical care, (3) increased household interpersonal conflict, and (4) feeling unsafe in their neighborhood.

**Methods:**

We used the 2020 California Health Interview Survey's AANHPI COVID Module, conducted weighted descriptive and multivariate analyses, and computed adjusted relative risks (RR). The multivariate models controlled for Asian subgroup, age, gender, immigrant status, education level, poverty, and English proficiency.

**Results:**

Among Asians who reported race-based hate (6.9% overall), 62.4% reported not experiencing race-based unfair treatment. Compared to Asians not reporting a hate incident, this “discordant” group was more likely to experience serious psychological distress (RR = 6.9), forgo necessary medical care (RR = 2.4), increased household interpersonal conflicts (RR = 2.7), and feel unsafe in their neighborhoods (RR = 3.0). The “concordant” group did not post significant effects for severe psychological distress nor forgoing necessary medical care.

**Discussion:**

Most Asians reporting hate did not report race-based unfair treatment, and this group is most affected by the consequences of a hate incident. We indicate future directions for research and policy.

## Introduction

During COVID-19, increased attention has focused on anti-Asian discrimination and its measurement, but research examining various methods used to measure discrimination and association with health outcomes is limited (1, 2). Hate and unfair treatment are related race-based discrimination that can be personally experienced or witnessed but do not completely overlap. Hateful events or actions include overt physical, or verbal abuse provoked by types of bias, microaggressions, or prejudice (3, 4). Conversely, unfair treatment or judgment is often synonymous with inequality or unfairness in a social setting. It may be seen as being passed over for a promotion (5), or receiving poor treatment when seeking care in a doctor's office (6) or when applying for social services (7). Regardless of the situation, the risk of poor health outcomes based on experiences of hate and/or unfair treatment is dependent upon the accumulating effect of the type of hate or unfair action, the volume and frequency of the experiences, the duration of time the events have had on the individual to both internalize and deteriorate morale, among other social moderating/determining factors (e.g., social support, resources, community resilience) (8). Under-reporting of unfair treatment may be driven by a variety of factors, including feelings of social desirability or emotion-focused coping shaped by normalizing scapegoating, xenophobia, colonial mentality, perpetual foreigner syndrome, the model minority myth, and other forms of anti-Asian racism fueled by popular media and codified by political and social institutions (9–12). Lack of self-efficacy and awareness, desire to avoid unwanted attention or embarrassment, fear of deportation or loss of work, and limited culturally-appropriate community resources to both report and seek intervention are a few barriers to reporting personal experiences and/or witnessing discriminatory events among minoritized and immigrant populations (13).

**Figure 1:**
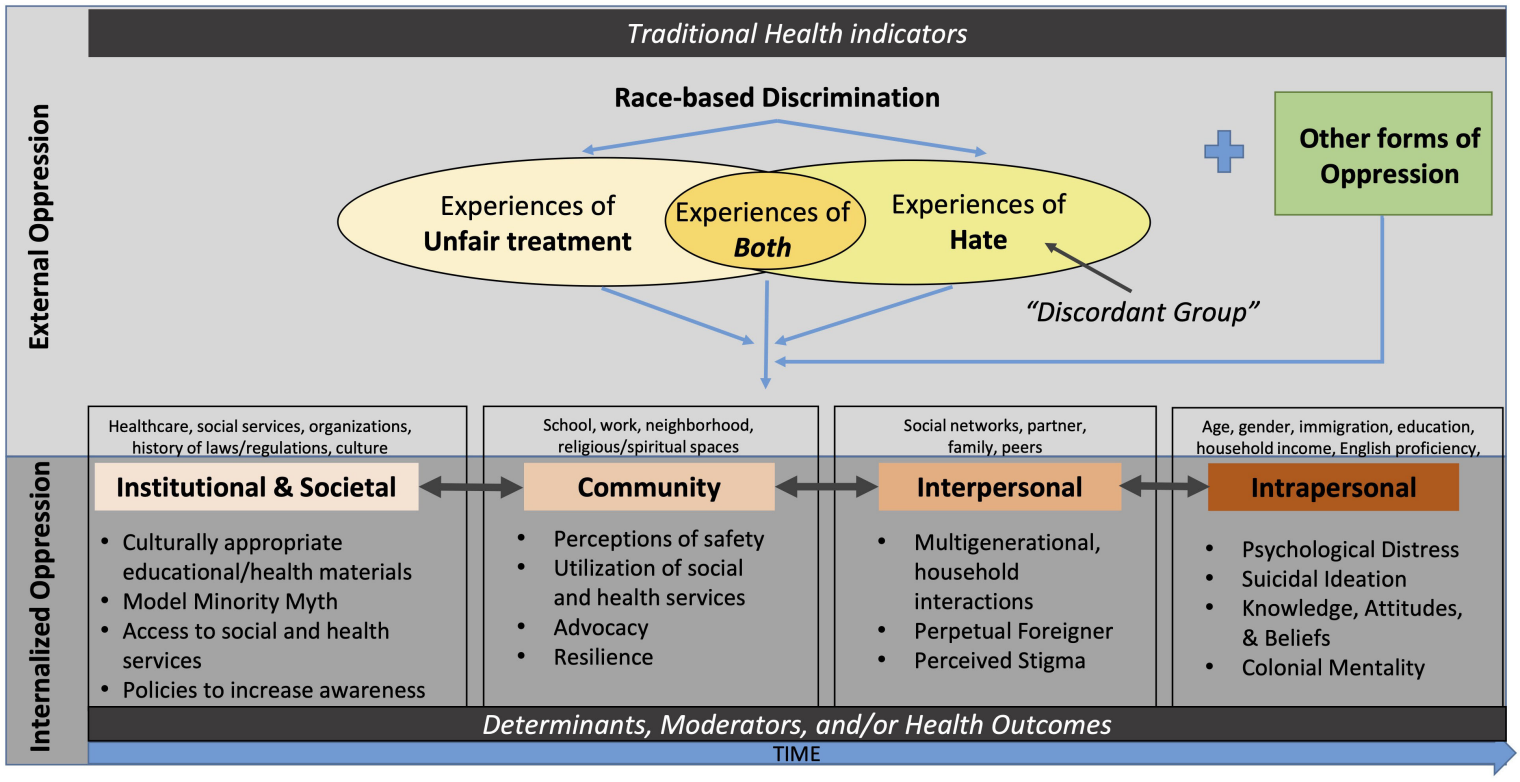
Conceptual framework on hate incidence experiences and health outcomes.

While not all who experience race-based unfair treatment may experience a hate incident, individuals who experience a hate incident may be expected to report experiencing unfair treatment based on their race; ideally, we would expect those to experience bias or prejudice-motivated threats to understand that such treatment is undeserved and wrong. Here, it is important to consider the deep generational history with varying levels of acceptance or tolerance throughout society, with further consideration of how race-based discrimination produces a cumulative impact within persons, communities, and societies over time (14). Among all victims experiencing the same event, many may not share similar feelings or attitudes toward their offenders, nor begin with the same level of acceptance or resilience due to sociodemographic, psychosocial, and previous discrimination differences. The overall impact of race-based discrimination is innately socioecological (e.g., a highly integrative system of societal factors that affect health), where one's demographics and actions (individual), relationships (interpersonal), living and working environments (community), and policies (societal) influence whether one feels well and thriving or desperate and declining (15). Figure 1 illustrates how one's experiences with discrimination also impacts their own mental health (individual), household or peer interactions (interpersonal), perception of safety (community), and healthcare experiences (structural). Although represented linearly, pathways are cyclical in Figure 1: it represents a visualization of race-based discrimination and multi-level health outcomes. Elements derived from race-based discrimination are contributors to systemic oppression such that societal norms and definitions, experiences of violent acts, and exercising dominance and power over another group not only perpetuates unjust treatment and hatred but also synergizes intersectionalities of inequity (e.g., race/ethnicity, gender, disability, nationality, sexual orientation, spirituality, immigration, etc.) (15, 16).

While understanding how anti-Asian discrimination impacts health is a critical need for Asian American health research, there is limited data available on these subjects, especially through large population health surveys. In response to this critical need during the pandemic, an Asian American and Native Hawaiian & Pacific Islander (AANHPI) COVID data module (17) was included in the 2020 California Health Interview Survey (CHIS), a population-representative state health survey (18). Co-developed with the UCLA Asian American Studies Center, the module includes questions on the frequency of hate incidents experienced and witnessed, and type of hate incidents. This module offers a unique measurement opportunity not found in other population surveys; experiences of a hate incident can be associated with a multitude of health outcomes, health access, and perceived neighborhood quality and safety outcomes. As part of the CHIS general survey, CHIS also collected data on unfair treatment due to race/ethnicity experienced during COVID-19. This question adds another dimension of anti-Asian discrimination experienced by the Asian community in California, the state with the greatest number (over 6 million) of the single race Asian population in the United States (19). Asians represent about 15% of the California population, compared with about 6% of the total US population (19).

In this paper, we are most interested in the “discordant” group of Asian adults who experienced a hate incident, but who reported not being treated unfairly. An individual could also report no hate incident experienced but report being treated unfairly. There are multiple domains of unfair treatment that do not manifest in directly experiencing a hate incident–such as physical or verbal abuse. The literature has established the effects of unfair treatment on health and mental health among Asian Americans (20), but the focal relationship of experiencing a hate incident is rarely measured in population health surveys and thus represents our study's new contribution. We posit that the reporting discordance of unfair treatment despite having experienced a hate incident may lead to greater internalizing behaviors associated with negative health behaviors and outcomes such as poor mental health, forgoing medical care, increased conflicts in the home, and lower perceived neighborhood safety.

## Methods

### Data and sample

To assess differential measurement of discrimination among the same research sample, we used the 2020 CHIS's AANHPI COVID-19 Module restricted file (17). This module was administered to CHIS adult respondents that reported any mention of “Asian” for race and conducted in English, Cantonese, Mandarin, Korean, Vietnamese and Tagalog. CHIS is a population-based multimode survey of California's residential, non-institutionalized population conducted every other year since 2001 and continually beginning in 2011. CHIS is one of the largest state-level health surveys representing over 20,000 households annually and collects information on adults, adolescents, and children (18). For more information on the main CHIS survey, please see www.chis.ucla.edu. CHIS typically surveys over 2,500 Asian adults in a given year, but since the AANHPI COVID-19 module was developed after the March 2020 stay-at-home orders in California and fielded beginning July 2020, the Asian sample in this study is a subset of the CHIS annual Asian sample (*n* = 700).

### Measures

Our choice of measures were guided by our conceptual framework (Figure 1) and measure availability in the AANHPI COVID-19 module restricted file.

#### Outcome variables

Serious psychological distress in the past month is a dichotomous variable based on the Kessler 6-item psychological Distress Scale (K6) with a maximum score of 24 and a minimum score of 0. “Serious levels of psychological distress” were scores at or above 13 and “no serious psychological distress” was defined as scores below 13.

Forgoing needed medical care in the past 12 months is a dichotomous variable indicating whether or not adults had to forgo necessary medical care. This variable was constructed using two CHIS questionnaire items. Individuals who reported that they delayed care in the past 12 months and that they did not get the care eventually were categorized as forgoing necessary care.

Increased household personal conflict is a constructed variable derived from the question, “During the stay-at-home orders connected to the COVID-19 outbreak, was there an increase in your household of any of the following?” that included the options, “interpersonal conflict with family members or loved ones,” “snapping at or yelling at family members or loved ones,” “physical punishment of family members or loved ones,” and “none of these.” Individuals who reported having “interpersonal conflict with family members or loved ones” were considered to have experienced increased household interpersonal conflict.

Feeling unsafe in neighborhood is a constructed variable derived from the question, “Do you feel safe in your neighborhood?” Individuals who reported feeling safe in their neighborhood “some of the time” and “none of the time” were categorized as feeling unsafe in their neighborhood.

#### Main predictor of interest

Our main predictor of interest is the extent of discordance of reporting experiencing hate and race-based unfair treatment. Reporting “discordance” is defined as reporting an experience of a hate incident, but not reporting an experience of unfair treatment. Reporting “concordance” is defined as reporting an experience of a hate incident and also reporting an experience of unfair treatment.

In the CHIS COVID-19 module, respondents were asked a multi-select question with a write-in option, “Have you experienced any of the following situations because of the coronavirus or COVID-19 outbreak?,” which included the option, “I've been treated unfairly because of my race/ethnicity.” Individuals who indicated this option were treated as having experienced unfair treatment due to their race/ethnicity. Respondents who identified as Asian, Native Hawaiian and/or Other Pacific Islander (NHPI) were also asked to respond to the statement, “Over the past 12 months, have you experienced any of the following situations because of the Coronavirus or COVID-19 outbreak?” followed up with the question, “I have directly experienced a hate incident due to the Coronavirus.” Individuals who responded affirmatively to the statement were treated as having experienced a hate incident in the past 12 months. Responses that were “not ascertained” and “I don't know” (*n* = 54; 7.7% of sample) were treated as zeroes and as neither experiencing unfair treatment nor experiencing a hate incident. For this analysis, we focused on Asian respondents.

#### Covariates

Asian subgroups were categorized as “East Asian,” “Southeast Asian,” “South Asian,” and “Other or Multiple Asian” using the NHPI and Asian subgroups variable. “East Asian” included individuals who identified as “Chinese,” “Japanese,” and “Korean.” “Southeast Asian” included individuals who identified as “Filipino,” “Vietnamese,” and “Southeast Asian.” “South Asian” included individuals who identified as “South Asian.” “Other or Multiple Asian” included individuals who indicated “other” or “belonging to two or more Asian subgroups.” Individuals who identified as “Native Hawaiian and/or Pacific Islander” only were excluded from analysis. Multiracial Asians were assigned to their single race Asian category they selected.

Age group was categorized using the survey vendor age continuous variable to reflect the following four categories: “18 to 25,” “26 to 39,” “40 to 64,” and “65 years old and older.”

Gender is a binary (male or female) constructed variable from the CHIS gender identity question, which accounts for “male,” “female,” “transgender,” or “none of these.” “Transgender” or “none of these” options are imputed as either “male” or “female” by the CHIS survey vendor.

English proficiency was defined from a CHIS 4-level variable redefined into a dichotomized variable: individuals who responded as “not well” or “not at all” were considered to have limited English proficiency, while individuals who responded “well” or “very well” were considered English proficient.

Immigrant status was assessed using the CHIS 3-level constructed variable with the following categories: “US-born citizen,” “Naturalized citizen,” and “Non-citizen.” An individual is considered an immigrant if they responded “Naturalized citizen” or “Non-citizen.”

Income as a percentage of the federal poverty level (FPL) was dichotomized using the FPL thresholds into “ <100% FPL” and “100% FPL and higher.”

Educational attainment was assessed using CHIS 9-level variable constructed into a dichotomized variable: “less than a bachelor's degree” and “bachelor's degree or higher.”

### Analysis

We conducted weighted descriptive and multivariate analyses, and computed post-estimation adjusted relative risks (RR). In our multivariate logit models, we assessed the association of reporting discordance on (1) serious psychological distress, (2) forgoing needed medical care (3) increased household interpersonal conflict during COVID-19, and (4) feeling unsafe in their neighborhood. The multivariate models were adjusted for Asian subgroup, age, gender, immigrant status, education level, poverty, and English proficiency. Significance was assessed at the alpha = 0.05 level. Sample weights were employed to account for complex sampling design and to calculate accurate variance estimations. All analyses were performed using Stata version 16.1.

## Results

Among Asian adults, 6.4% reported experiencing a hate incident and 4.4% of Asians reported being unfairly treated due to race or ethnicity. Of those who reported experiencing a hate incident, a majority (62.4%) reported not experiencing unfair treatment due to race or ethnicity. Table 1 displays sample characteristics stratified by the discordant group, concordant group, and the group that did not report experiencing a hate incident. Within the discordant group, 22.1% reported high levels of psychological distress, 25.0% reported forgoing necessary medical care, 23.5% reported increased household interpersonal conflicts, and 28.0% reported feeling unsafe in their neighborhood. A majority of the discordant group identified as Southeast Asian (72.7%), despite the fact that Southeast Asians represented only 39% of the overall sample. About half of the discordant group (50.3%) were between the ages of 40 to 64 years old. Most of discordant group members identified as female (62.6%), were English proficient (94.1%), identified as an immigrant (69.3%), reported an income at or above 100% FPL (85.5%), and obtained a bachelor's degree or higher (70.6%).

**Table 1:** Hate incidents experienced and unfair treatment, Asian adults, California health interview survey AANHPI COVID-19 module 2020.

	**Experienced a hate incident**				
	**All**		**Discordant**	**Concordant**	**No hate incident experienced**
	**Observations**	**Weighted %**	**Weighted %**	**Weighted %**	**Weighted %**
**Serious psychological distress**					
Yes	37	5.6%	22.1%	29.0%	4.3%
No	663	94.4%	77.9%	71.0%	95.7%
**Forgone needed medical care**					
Yes	67	9.9%	25.0%	15.4%	9.2%
No	633	90.1%	75.0%	84.6%	90.8%
**Increased household interpersonal conflicts**					
Yes	90	12.5%	23.5%	46.3%	11.1%
No	610	87.5%	76.5%	53.7%	88.9%
**Feeling unsafe in neighborhood**					
Yes	64	10.2%	28.0%	0.0%	9.7%
No	636	89.8%	72.0%	100%	90.3%
**Asian subgroup**					
East Asian	335	43.2%	23.8%	69.4%	43.4%
Southeast Asian	266	39.0%	72.7%	17.2%	38.1%
South Asian	64	13.6%	0%	13.3%	14.2%
Other Asian/Two or more Asian	35	4.1%	3.5%	0.0%	4.3%
**Age group**					
18–25	71	14.0%	8.1%	55.2%	13.2%
26–39	175	25.0%	11.3%	19.1%	25.8%
40–64	307	42.2%	50.3%	23.7%	42.3%
65+	147	18.8%	30.3%	2.0%	18.7%
**Gender**					
Female	375	53.4%	62.6%	57.7%	52.9%
Male	325	46.6%	37.4%	42.3%	47.1%
**English proficiency**					
Limited	82	16.0%	5.9%	0.0%	16.9%
Proficient	618	84.0%	94.1%	100%	83.1%
**Immigrant status**					
Immigrant	466	72.0%	69.3%	55.4%	72.6%
Not an immigrant	234	28.0%	30.7%	44.6%	27.4%
**Income as % FPL**					
< 100% FPL	66	11.2%	14.5%	11.9%	11.0%
100% FPL and higher	634	88.8%	85.5%	88.1%	89.0%
**Educational attainment**					
BA/BS or higher	491	60.2%	70.6%	78.3%	59.3%
Less than BA/BS	209	39.8%	29.4%	21.7%	40.7%
Observations	700		33	14	653

“Other Asian/Two or more Asian” category includes single race groups that responded “yes” to Asian, but “Other” in the subgroup follow-up question and two or more Asian groups. Multiracial Asians were assigned to the single race Asian category.

Table 1 further shows that within the concordant group, 29.0% experienced serious psychological distress, 15.4% forgone necessary medical care, and 46.3% experienced increased household interpersonal conflicts. No participants within the concordant group reported feeling unsafe in their neighborhood. A majority of the concordant group identified as East Asian (69.4%), though East Asians only represented 42.2% of the overall sample. A majority of the concordant group identified as ages 18–25 (55.2%), identified as female (57.7%) and an immigrant (55.4%), had an income at or above 100% FPL (88.1%), and obtained a bachelor's degree or higher (78.3%). All respondents in the concordant group reported high English proficiency (100.0%). Sample characteristics displaying row percentages by extent of discordance for each outcome and covariate can be found in the Appendix.

Table 2 shows the results of the multivariate logistic regression models. Compared to Asian adults who did not report experiencing a hate incident, the discordant group was 6.9 times more likely to experience severe psychological distress (RR = 6.93; *p* < 0.001), 2.7 times more likely to forgo necessary medical care (RR = 2.69; *p* = 0.014), 2.7 times more likely to experience increased household interpersonal conflicts (RR = 2.66; *p* < 0.001), and 3.5 times more likely to feel unsafe in their neighborhood (RR = 3.48; *p* < 0.001), on average, holding all else constant.

**Table 2:** Relative risk of selected outcomes by discordant reporting of hate incident experience and unfair treatment, Asian adults, California health interview survey AANHPI COVID-19 module 2020.

	**Severe psychological**			**Forgone necessary**			**Increased household**			**Feeling unsafe**		
	**distress**			**care**			**interpersonal conflicts**			**in neighborhood**		
	**RR**	***p*** **-value**	**[95% CI]**	**RR**	***p*** **-value**	**[95% CI]**	**RR**	***p*** **-value**	**[95% CI]**	**RR**	***p*** **-value**	**[95% CI]**
**Discordance**																
Discordant	6.93	< 0.001^**^	[3.30, 14.54]	2.69	0.014^*^	[1.23, 5.91]	2.66	< 0.001^**^	[1.50, 4.70]	3.48	< 0.001^**^	[1.80, 6.72]
Concordant	3.00	0.121	[0.75, 12.05]	1.37	0.694	[0.29, 6.43]	2.58	0.014^*^	[1.21, 5.49]	—	—	—
No hate incident	1			1			1			1		
**Asian subgroup**																
East Asian	1			1			1			1		
Southeast Asian	1.18	0.660	[0.57, 2.46]	0.69	0.270	[0.36, 1.33]	0.41	0.002^*^	[0.23, 0.71]	0.71	0.219	[0.41, 1.23]
South Asian	1.08	0.912	[0.28, 4.15]	0.82	0.689	[0.32, 2.14]	0.83	0.565	[0.43, 1.58]	1.98	0.067	[0.95, 4.11]
Other Asian/Two	1.27	0.731	[0.32, 5.03]	0.85	0.810	[0.24, 3.08]	0.76	0.541	[0.31, 1.85]	0.53	0.405	[0.12, 2.36]
or more Asian
**Age group**																
18–25	11.66	< 0.001^**^	[4.22, 32.20]	1.13	0.805	[0.44, 2.92]	1.20	0.562	[0.65, 2.21]	0.69	0.437	[0.28, 1.75]
26–39	3.11	0.016^*^	[1.24, 7.82]	0.27	0.010^*^	[0.10, 0.73]	0.88	0.621	[0.52, 1.47]	0.54	0.129	[0.24, 1.20]
40–64	1			1			1			1		
65+	0.77	0.759	[0.15, 4.06]	0.83	0.636	[0.37, 1.83]	0.04	< 0.001^**^	[0.01, 0.18]	0.65	0.360	[0.26, 1.64]
**Gender**																
Female	1.10	0.814	[0.50, 2.42]	1.95	0.034^*^	[1.05, 3.62]	1.41	0.132	[0.90, 2.20]	0.99	0.971	[0.58, 1.69]
Male	1			1			1			1		
**English proficiency**																
Limited	2.10	0.115	[0.84, 5.29]	1.37	0.452	[0.60, 3.15]	0.40	0.221	[0.09, 1.73]	1.83	0.185	[0.75, 4.45]
Proficient	1			1			1			1		
**Immigrant status**																
Immigrant	0.96	0.920	[0.40, 2.29]	0.78	0.491	[0.39, 1.58]	0.44	< 0.001^**^	[0.28, 0.70]	0.68	0.255	[0.35, 1.32]
Not an immigrant	1			1			1			1		
**Income as % FPL**																
<100% FPL	0.45	0.295	[0.10, 1.99]	0.74	0.526	[0.29, 1.89]	0.70	0.383	[0.31, 1.57]	2.19	0.059	[0.97, 4.95]
100% FPL and higher	1			1			1			1		
**Educational attainment**																
BA/BS or higher	0.78	0.497	[0.38, 1.61]	0.88	0.699	[0.47, 1.65]	0.72	0.257	[0.41, 1.27]	0.63	0.200	[0.31, 1.28]
Less than BA/BS	1			1			1			1		
Observations	700			700			700			686		

* p < 0.05,

** p < 0.001.

“Other Asian/Two or more Asian” category includes single race groups that responded “yes” to Asian, but “Other” in the subgroup follow-up question, as well as two or more Asian. The association between Concordant group with Feeling Unsafe in Neighborhood predicted failure perfectly, therefore these 14 observations were dropped. FPL, Federal Poverty Level.

The concordant group was found to be 2.6 times more likely to have increased household interpersonal conflicts (RR = 2.58; *p* = 0.014) compared to individuals who did not report experiencing a hate incident, on average, holding all else constant. The concordant group did not post statistically significant effects for severe psychological distress (*p* = 0.121) nor forgoing necessary medical care (*p* = 0.694) compared to the group who did not report experiencing a hate incident. There were zero observations in the concordant group who reported feeling unsafe in their neighborhood.

## Discussion

During the course of the COVID-19 pandemic in 2020, our study found that most Asians reporting a hate incident did not report race-based unfair treatment, and it is this group that is most affected by the harmful consequences of experiencing a hate incident, psychologically and socially. This suggests a significant discordance between respondents' reporting of experiencing hate incidents and recognition of unfair treatment based on race/ethnicity. The concordant and discordant groups showed similar effect sizes for increased interpersonal conflicts in the multivariable models, but the discordant group reported significantly worse severe psychological distress, forgoing of necessary care, and feeling unsafe in their neighborhoods when compared to Asians not experiencing a hate incident. The factors that drive the disconnect between experiences of hate incidents and lack of recognition of discriminatory events as unfair treatment, the social context that allow such factors to develop in the first place, and the impacts of this reporting discordance on health and wellbeing, altogether, represent important concepts to assess further.

In our sample, the discordant group vs. the concordant group displayed several key demographic differences worth further exploration and explanation. The discordant group had higher percentages of Southeast Asians, respondents ages 40–64 and 65+, and respondents experiencing poverty. The concordant membership included higher percentages of East Asians, respondents ages 18–25, and respondents at or above 100% FPL. These represent intersectionalities that have effects that may be missed by traditional regression analysis. We posit that sociopolitical conditions, migration patterns, cultural norms for coping (e.g., adaptive skills, internalization) and mental health (e.g., stigma), and intergenerational effects may be moderating drivers for the discordant group. For example, young adults may be more keenly aware of the various types of discrimination and become more empowered by their social networks to report or reflect on their experiences. Conversely, older adults may have developed maladaptive coping mechanisms compounded overtime, which may have resulted in further detachment and passive coping skills. Regardless of discordance or concordance, in this population-based sample, gender-based differences were observed in reports of experiencing a hate incident (53.4% females vs. 46.6% males), which is consistent with Stop AAPI Hate reports where over 63% of hate incidents were reported by women (21). Importantly, our study shows a higher proportion of females vs. males are in the discordant group, suggesting possible under-reporting of hate incidents inflicted against Asian women. Due to gendered racialization, the intersectional experiences of Asian women subject them to increased oppression and marginalization (e.g., hyper-sexualization, fetishization, stereotypic depictions of subservience and passiveness), rendering them invisible and disposable in the broader American racial hierarchy. Furthermore, all concordant group members reported feeling safe in their neighborhood, perhaps implicating the role of social support and community connectedness in buffering against the deleterious effects of race-based hate and in empowering others to actively recognize unfair treatment, which coincides with cognitive theorists' hypotheses that associate social support and psychological resources with lesser discrimination-related stress (22, 23).

Existing theories from the public health literature are worth considering to build plausible explanations and models around Asian discrimination discordance. Minority stress theory (24) provides a conceptual framework on how experiences of discrimination (i.e., external oppression) and internalized negative feelings around one's own minority group or identity (i.e., internal oppression) promote poor mental health issues among people of color and minoritized groups. One pathway (25, 26) suggests that external oppression leads to psychological distress by internalized oppression. Inspired by multicultural-feminist scholars (27), another association posits multiple oppressions (e.g., racism and xenophobia, racism and colonialism, racism and sexuality) fuse together to form individual's experiences of discrimination, manifesting as poor mental health or other inadvertently self-deprecating or self-sabotaging behaviors (e.g., delaying or avoiding health care services). All types of hate and unfair treatment, along with other forms of oppressed experiences exist, operate, and reproduce each other across and within the various socioecological levels to cause harmful and negative outcomes (Figure 1).

In this current study, it is possible that the discordant group may be experiencing higher levels of serious psychological distress than the concordant group due to higher utilization of avoidant coping strategies, such as tolerating years or high frequency of unfair treatment as it relates to moral injury or distress often experienced by among minoritized or racialized individuals (8). Race-based traumatic stress theory, derived from counseling psychology, indicates that racial and ethnic minorities experiencing racial discrimination may evoke symptoms and reactions comparable to that of post-traumatic stress disorder (28, 29). Trauma-exposed individuals are more likely to engage in passive and/or avoidant coping strategies to manage overwhelming distress through cognitive avoidance or emotional numbing. Studies reveal that those with passive and/or avoidant coping strategies in response to racial discrimination (e.g., keeping it to themselves or normalizing not reporting it) demonstrate deleterious impacts on negative psychological health and increased dissociative symptoms (i.e., momentary memory lapses memory or awareness; surrounding distortions) (30, 31). Given that the discordant group were more likely to report feeling unsafe in their neighborhood and forgoing necessary medical care, they may subscribe to avoidant strategies in the absence of social and community support, especially from employer leadership during the pandemic.

Moreover, the learned helplessness hypothesis (32) suggests that individuals continuously exposed to aversive stimuli may become conditioned to believe that their negative situations are uncontrollable or inescapable, resulting in an unwillingness to try and change their circumstance. It is possible that individuals in our discordant group were exposed to racial discrimination and other forms of oppression resulting in feelings of helplessness and a resignation to resolve these negative situations due to no direct, positive impacts for the victim. Feelings of helplessness may then mediate the relationship between racial discrimination and elevated mental distress (33).

However, beyond these psychological and public health theories, it is imperative that researchers engage with other social science fields (i.e., history, sociology, anthropology, and ethnic studies), which are rich in theoretical backgrounds that may fill gaps to better understand anti-Asian racism. For example, Asian American studies and ethnic studies can provide clarity into the fuller context (e.g., social, political, cultural, economic, historical, etc.) around anti-Asian hate to better inform health-related work. Greater recognition of the importance of ethnic studies in understanding health outcomes are already taking place as it pertains to health education and health policy (34), and similar perspectives would also apply to the development of research questions and interpretations of analyses. Relevant to the work here, prior work from Asian American Studies examined anti-Asian violence using a variety of qualitative and thematic perspectives, including racial categorizing and racialization of Asians, nativism, patriotic racism, racial hierarchization, and subsequent interracial conflict (35). As such, if we aim to understand various pathways into discordant behavior as well as contribute substantively to broader academic work on anti-Asian hate, active engagement with other fields is not only a complementary addition to this evolving work, but increasingly necessary to dismantling structural racism.

Moving beyond the academic and into policy considerations, the state's ability to appropriately capture anti-Asian sentiment in public data as well as adequately direct resources that provide deterrence and mitigation is under criticism both, before and during COVID-19, leading non-governmental efforts to document hate-motivated behaviors (21). While efforts like Stop AAPI Hate are critical for recognizing increased burdens of hate incidents, public statistics collected by state and federal organizations carry unique weight, including in broadly guiding recognition of these issues and supporting as well as securing funding and investment. However, there are a few notable issues with Asian hate crime reporting. Asians are less likely to report hate crimes compared to other victims. Prior work has also demonstrated substantial misalignment between experience of hate-motivated behavior and actual recognition of hate crimes in federal databases, driven by not only gaps in reporting of incidents to police but subsequent registration of hate crime incidents by police (4, 13, 36). There is also some conceptual differences between experiences of anti-Asian violence and discrimination vs. the actual classification of such events as bias incidents vs. hate crimes, adding further complexity to discussions about how anti-Asian sentiment manifests itself as distinct occurrences of discrimination. Our work provides an approximation of the magnitude of the under-reporting of hate crimes: of Asian adults who said they experienced a hate incident, about 62% did not declare that they had experienced unfair treatment. Without the recognition of the hate incident as unfair treatment, we suggest that individuals may not feel compelled to take action against the hate incident by reporting it to the police. Several factors described earlier (lack of awareness, normalizing scapegoating, desire to avoid unwanted attention) serve to explain the potential reasons why Asians are not recognizing these hate incidents as unfair (13).

### Limitations

There are a few limitations of this research. First, these analyses are cross-sectional and interpretations are associations, not casual links. Second, the question on unfair treatment was not conditional on reporting experiencing a hate incident, although the questions were adjacent in the CHIS COVID-19 modules. Third, the question on experiencing a hate incident did not directly ask if it was due to race/ethnicity; however, this question was asked during a timeframe in which race-based discrimination and hate crimes toward Asians were on the rise. Second, this study was conducted only in California. Patterns of anti-Asian hate as well as subsequent impacts may differ in other states. Third, given that the AANHPI COVID-19 module was administered in the middle of 2020 following the initial public health declarations of COVID-19 as a pandemic, the CHIS 2020 sample size used was limited, providing a key barrier in generating insights for Asian subgroups, an important component of appropriately contextualizing health needs (11, 12, 37). While preliminary results showed differences among East and Southeast Asians, subgroup-based analyses will provide far richer insights based on each individual group's needs.

The 2021 AANHPI COVID module will be available in the fall of 2022 with an expected sample of around 4,000 adults. Further work should examine the association of experiences of hate and unfair treatment with outcomes with increased sample size to ensure appropriate analytical power for Asian subgroup analyses. Other work should consider the ways the state and federal government collect data on anti-Asian hate, which mostly consists of recording hate crimes, as well as how current infrastructures fail to recognize, mitigate, and deter anti-Asian hate. Asians are less likely to report hate crimes to the police (13); this fact is a key driver and consequence of public policies doing too little or too late in appropriately combating anti-Asian hate.

While state resources should be invested in more comprehensive public reporting infrastructure, investment in community organizations that support minorities and Asians should be considered vital for combating anti-Asian racism. Community organizations and/or religious institutions play an important, supportive role in the lives of their ethnic members on an everyday basis and, indeed, in the aftermath of a hate incident. California could serve as a model with its $156.5 million investment to measure and address systemic discrimination and rising hate incidents against AAPI communities. On July 13, 2021, Governor Newsom signed the historic Asian and Pacific Islander (API) Equity Budget, sponsored by the California API Legislative Caucus. Investments include data collection efforts (including continuing to support an AANHPI module in CHIS in 2022), and community-based services to address systemic racism and discrimination in the AANHPI communities. Federally and for other states, California's explicit agenda to stop anti-Asian hate should be modeled and upheld in advocacy, research and policy-making (38).

## Data availability statement

The data analyzed in this study is subject to the following licenses/restrictions: The dataset is a restricted file that can be accessed by an application to the CHIS Data Access Center. Requests to access these datasets should be directed to dacchpr@em.ucla.edu.

## Ethics statement

The studies involving human participants were reviewed and approved by the UCLA IRB Data Access Center Protocol: Title: California Health Interview Survey Data (CHIS) Data Access Center (DAC) Number: IRB#11-002227. The patients/participants provided their written informed consent to participate in this study.

## Author contributions

NP, AA, and RB conceptualized the manuscript. NP acquired access to the restricted AANHPI COVID-19 module file and led the study design and statistical analyses supported by RB and ST. MS-L, RB, AA, and NP contributed to the introduction and discussion sections. MS-L led the development of the conceptual framework and RB and ST led the write-up of the methods and results section. All authors co-edited, reviewed, and approved the final manuscript.

## Funding

NP and ST report support from UCLA Asian American Studies Center and the CHIS funders who supported the general COVID-19 Module (PI Ponce)– The California Wellness Foundation, the California Health Care Foundation, the California Endowment and the California Department of Public Health. In addition the NIH Community Engagement Alliance (CEAL) Against COVID-19 Disparities Program (PI: Arleen Brown) supported the CHIS AANHPI COVID-19 monthly data dashboards. MS-L reports support from the NIMHD/NIH RADx-UP (U01MD017434, mPI: MS-L and Kwan). RB reports support from the Agency for Healthcare Research and Quality as part of the Los Angeles Area Health Services Research Training Program (T32 Predoctoral Fellowship) during the conduct of this study.

## Conflict of interest

At time of submission, AA was employed by Precision Advisors, a for-profit healthcare consulting firm. The remaining authors declare that the research was conducted in the absence of any commercial or financial relationships that could be construed as a potential conflict of interest.

## Publisher's note

All claims expressed in this article are solely those of the authors and do not necessarily represent those of their affiliated organizations, or those of the publisher, the editors and the reviewers. Any product that may be evaluated in this article, or claim that may be made by its manufacturer, is not guaranteed or endorsed by the publisher.
