# Supplementary material for: Measuring Asian hate: Discordant reporting of race-based hate incidents and unfair treatment and association with measures of wellbeing
Source: Front Public Health. 2022 Oct 10;10:958857. doi: 10.3389/fpubh.2022.958857 (PMC9589279; doi:10.3389/fpubh.2022.958857)
Supplement: Supplementary file 1 [file Table_1.DOCX]

Appendix. Hate Incidents Experienced and Unfair Treatment, Asian Adults,

California Health Interview Survey AANHPI COVID-19 Module 2020

|  |  |  |  |
| --- | --- | --- | --- |
|  | **Experienced a Hate Incident** | |  |
|  | **Discordant** | **Concordant** | **No Hate Incident Experienced** |
|  | Weighted % | Weighted % | Weighted % |
| Serious Psychological Distress |  |  |  |
| Yes | 15.6% | 12.3% | 72.2% |
| No | 3.3% | 1.8% | 94.9% |
| Forgone Needed Medical Care |  |  |  |
| Yes | 10.0% | 3.7% | 86.3% |
| No | 3.3% | 2.3% | 94.4% |
| Increased Household  Interpersonal Conflicts |  |  |  |
| Yes | 7.5% | 8.9% | 83.6% |
| No | 3.5% | 1.5% | 95.1% |
| Feeling Unsafe in Neighborhood |  |  |  |
| Yes | 10.9% | 0.0% | 89.1% |
| No | 3.2% | 2.7% | 94.2% |
| Asian subgroup |  |  |  |
| East Asian | 2.2% | 3.9% | 94.0% |
| Southeast Asian | 7.4% | 1.1% | 91.5% |
| South Asian | 0.0% | 2.3% | 97.7% |
| Other Asian/Two or more Asian | 3.3% | 0.0% | 96.7% |
| Age Group |  |  |  |
| 18-25 | 2.3% | 9.5% | 88.2% |
| 26-39 | 1.8% | 1.8% | 96.4% |
| 40-64 | 4.7% | 1.3% | 93.9% |
| 65+ | 6.4% | 0.3% | 93.3% |
| Gender |  |  |  |
| Female | 4.7% | 2.6% | 92.8% |
| Male | 3.2% | 2.2% | 94.6% |
| English Proficiency |  |  |  |
| Limited | 1.5% | 0.0% | 98.6% |
| Proficient | 4.5% | 2.9% | 92.7% |
| Immigrant Status |  |  |  |
| Immigrant | 3.8% | 1.8% | 94.3% |
| Not an Immigrant | 4.4% | 3.8% | 91.8% |
| Income as % FPL |  |  |  |
| Less than 100% FPL | 5.2% | 2.5% | 92.3% |
| 100% FPL and higher | 3.8% | 2.4% | 93.8% |
| Educational Attainment |  |  |  |
| BA/BS or higher | 4.7% | 3.1% | 92.2% |
| Less than BA/BS | 2.9% | 1.3% | 95.8% |
| Observations | 33 | 14 | 653 |

“Other Asian/Two or more Asian” category includes single race groups that responded “yes” to Asian, but “Other” in the subgroup follow-up question and two or more Asian groups. Multiracial Asians were assigned to the single race Asian category.
